# 

**Published:** 1882-01

**Authors:** 


### INDEX.

PAGE.
Abdominal dropsy complicating pregnancy........ 65
Acute articular rheumatism........ 68
jaundice in sheep........ 51
Agricultural society, royal........ 268
Allen, Thomas A.—tuberculosis in a cow........ 258
American public health association........ 50
veterinary college commencement........ 174
Animals frozen, resuscitation of........ 186
burial grounds for........ 200
domestic, diseases to which are most subject........ 155
some things that happen to our........ 156
insanity in the lower........ 309
respiration of lower........ 85
that drink rum........ 269
the contagious diseases of domestic........ 13
Apoplexy, puerpural........ 43
Arstingstall, George—breeding of elephants in captivity........ 146
Arthritis, purulent........ 245

Bear, convulsions in........ 70
Bears of Berne........ 195
Beres, H.A.—acute articular rheumatism secondary to catarrhal influenza. 68
Berns, George H.—epizootic suppurative cellulitis........ 29
Bird, a wonderful........ 95
Bladder, scirrhous carcinoma of the........ 58
Blood sweating........ 57
Bovine post mortem........ 337
Bowman, E. P.—probable carcinomatous tumors encroaching upon the alimentary tract........ 69
Breder, Edward S.—strongylus, seu eustrongylus gigas ascaris renalis.... 168
Bronchiectasy in a calf........ 317
Bronchitis, chronic........ 319

Calf, hair balls in a........ 264
Calves, diptheria in........ 266
Camel's lung........ 320
Caries of a molar tooth........ 323
Cartilages, nutritive changes in the........ 119
Case department........ 55, 161, 255, 317

Caton, John Dean—observations on the development of special senses... 109
Cats, cemetery for........ 270
effect of arsenic on........ 266
Cattle law, Wyoming........ 192
lung sickness of........ 192
plague........ 199
remedy for........ 190
pleuro-pneumonia in........ 186, 189
quarantine........ 335
restaurants........ 93
size of spleen in........ 84
Spanish........ 194
temperature of........ 84
troughs........ 196
unknown disease among........ 334
Cellulitis, epizootic suppurative........ 29
Charbon, protection of cattle against........ 79
Chloral *vs.* strychina........ 86
Chloroform, a new mode of treatment with........ 87
Circulatory organs, some anomalies of the........ 157
Colic, a case of........ 259
in horses........ 48
Colites, preceeded by acute diarrhœa........ 67
Columbia Veterinary College, commencement........ 173
Comparative pathology, the study of........ 1
Conklin, William A.—catarrhal phthisis in a llama........ 321
degenerating hæmatoma in an iguana........ 322
grando or chalazion in an eagle........ 322
Contagion, pet animals as carriers of........ 278
Contagious diseases of domestic animals, the........ 13
Controlling sex........ 79
Convulsions in a bear........ 70
Correspondence........ 72, 172
Cow, acute lead poisoning in a........ 257
cæsarian section in a........ 268
kidney disease in the........ 265
Cow's brain, weight of........ 194

Diarrhœa amongst calves........ 191
Digestion in rumanants........ 235, 252
physiology of........ 136
Discourteous treatment of an expert witness........ 53
Disease, stamping out........ 278
the salmon........ 276
Diseases among Texas cattle........ 82
of the foot........ 303
the contagious, of domestic animals........ 13
to which domestic animals are most subject........ 155
Disinfecting agents, best........ 186

Dog, bile pigments in the urine of the........................ 265
disease in the arctic regions........................ 82
fasting........................ 89
pachymeningitis in a........................ 264
nose bleeding in........................ 265
Do Southern hogs have trichinæ........................ 50
Dromedary, suppurative nephritis in........................ 256
Dropsy, abdominal, complicating pregnancy........................ 65
Duck-rearing in China........................ 273

Eagle, grando or chalazion in an........................ 322
Editorial department........................47, 154, 249, 309
Elephant, cutaneous disease of the........................ 301
breeding in captivity........................ 146
diseases of........................ 158
English veterinary degrees........................ 52
Epizootic, suppurative cellulitis........................ 29
Equine cemeteries........................ 53
Erysipelas, phlegmonous, in horses........................ 29
Expansion and contraction of the foot and how to shoe it........................32, 225
Exports and imports from New York........................ 96
Eye, foreign bodies in the anterior chamber of the........................ 115
Farmers' and dairymens' association........................ 177
Fever, milk........................ 63
Fishes, digestion of........................ 81
Flesh and fat producers........................ 78
Foot, expansion and contraction of the........................ 32
some diseases of the........................ 303
Freak of nature, a........................ 279
Free-martins........................ 201
Frey, Mark L.—colitis, preceded by acute diarrhœa........................ 67

Gastro-entoritis, acute........................ 258
Glanders........................ 193
the test for........................ 312
Goat society, the........................ 197
some diseases of the........................ 130
Goitre in the lower animals........................ 265
Greenwood cemetery, report........................ 194
Hæmato-pyo-metra in a bitch........................ 320
Haygard, E. T.—abdominal dropsy complicating pregnancy........................ 65
Herbivora, a blood diet for........................ 330
Hippopotamus, the........................ 97
Hog, a hemaphrodite........................ 276
cholera, antidote for........................ 77
Hogs, do southern, have trichinæ........................ 50
Homœopathy in veterinary practice........................ 47
Horse, a double-faced........................ 253
cruelty to a........................ 273

Horse, disease, new.... 195
flatulent colic in a.... 260
gall stone in a.... 264
impaction of the color of a.... 260
larcerated wound of the neck in a.... 261
teeth of, and their diseases....143, 288
the photographer of the.... 199
typhoid fever in a.... 264
with a grievance.... 335
Horses, advance in the price of.... 92
a lecture on.... 269
Arab.... 337
Australian wild.... 96
colic in, cause of death.... 48
Clydesdale.... 195
curing balky.... 197
experiments with blood meal on.... 265
feeding.... 332
finding their way home.... 91
hœmoglobinuria in.... 254
jibbing.... 97
morphine in the colic of.... 314
new cause for bloody urine in.... 251
phlegmonous erysipelas in.... 29
recent discoveries of fossil.... 281
scarlet fever among.... 195
street car, how fed.... 196
to keep flies from.... 271
Turkish baths for.... 272
white.... 90
foot, the contraction and expansion of the.... 225
frog, the.... 189
hoof expand, how much does the.... 251
tail, bird's nests in a.... 270
Horse-cleaning machine.... 269
flesh, consumption of....92, 269
Hunt, Ezra M.—practical suggestion to young veterinarians.... 241
the contagious diseases of domestic animals.... 13
Hydrophobia and its cure....80, 315, 339
notes on.... 188
precautions against.... 77

Iguana, degenerating hæmatoma in an.... 322
Influences of parents.... 93
race upon the action of poison.... 275
Insanity in the lower animals.... 309
Intussusception of the ilium.... 255

Japanese food.... 92

Jaw of the crocodile and dog, muscular strength of........................ 81
Jennings, R.—the teeth of the horse and their diseases...............143, 288

Laparo-hysterotomy........................................................ 70
Lice, remedy for.......................................................... 265
Lindsay, J.—rumenotomy.................................................... 164
Literary notes............................................................ 315
Live stock in the United States........................................... 277
Llama, catarrhal phthisis in.............................................. 321
Lupins, poison in......................................................... 89

Margosse oil.............................................................. 90
Massage in veterinary therapeutics........................................ 311
Mercury, effect of minute doses on animals................................ 78
Metrotomy................................................................. 171
Milk fever................................................................ 63
secretion, effect of medicines upon....................................... 250
vein...................................................................... 186
ways of which, may be effected............................................ 270
Mitchell, Hubbard W.—a lesson in comparative zoology...................... 292
Monkeys................................................................... 201
effects of cod-liver oil on............................................... 187
Monstrosities.............................................................253, 278
Montreal Veterinary College, commencement................................. 175
Moore, William Oliver—foreign bodies in the anterior chamber of the eye. 115
Mule, a breeding mare..................................................... 79

Navin, John N.—sudor cruentus or blood sweating........................... 57
the expansion and contraction of the horse's foot and how to shoe it......32, 225
Nephritis, suppurative, in a young dromedary............................... 256
News and miscellany.......................................89, 194, 268, 332
Newton J. V.—metrotomy.................................................... 171
tenatomy.................................................................. 172
Notices................................................................... 159

Obituary.................................................................. 327
Œsophagus, ulcer of the................................................... 170
Oil cake.................................................................. 90
meals..................................................................... 186
Ontario Veterinary College, commencement.................................. 175
Osler, William, and A.W. Clement—diffuse purulent bronchiectasy in a calf. 317
Ostrich farming........................................................... 198

Paralysis in cows......................................................... 254
Parasites, trichinæ like................................................... 86
Parkinson, George M.—acute gastro-enteritis from an overdose of sodium chloride.... 258
acute lead poisoning in a cow............................................. 257

Parkinson, George M.—a novel way of feeding pups.................... 261
intussusception of the ilium in a two-year-old stallion.................................... 255
scirrhous carcinoma of the bladder............. 58
Parrot, calcified arteries in a.................................... 264
Pasteurs vaccinations........................................... 78
Pathology, claims of comparative.................................. 94
Pigeons, food for carrier.......................................... 77
Porter, William Henry—mixed central sarcoma of the left humerus...... 55
pathology of the nutritive changes in the cartilages and of acute synovitis........................... 119
purulent arthritis................................. 245
Pregnancy in a quail, extra uterine................................ 83
Proceedings of societies........................................... 326
Progress of veterinary science.....................77, 186, 264, 330
Public health association.......................................... 91
Puerpural apoplexy.................................................. 43
Pulse, the............................................................ 332
Python eggs.......................................................... 274
gangrena oris in a ........................................ 255

Rabbits, contagious pyæma of....................................... 86
Rabies, a possible cause and probable preventive.................... 77
Reports of societies................................................ 173
Resuscitation of animals............................................ 266
Reticulated round-cell sarcoma...................................... 167
Reviews...........................................74, 179, 262, 328
Rheumatism, acute articular......................................... 68
Robertson, Thomas—puerpural apoplexy................................ 43
Rumenotomy.......................................................... 164

Salmon disease and its lessons..................................... 331
Satterthwaite, Thomas E.—the study of comparative pathology.......... 1
tuberculosis in men and animals..........101, 203
Sarcoma of the left humerus......................................... 55
orbit................................................... 60
Schmidt, Max—the care of cutaneous diseases of the elephant in captivity. 301
School, a new veterinary............................................ 91
Scirrhous carcinoma of the bladder.................................. 58
Scratches, treatment of............................................. 159
Seers, B. C.—two cases diagnosticated as milk fever................. 63
Senses, development of special...................................... 109
Sewall, A. J.—some diseases of goats................................ 130
Sheep, acute jaundice in............................................ 51
decrease of, in England...................................... 268
dip, recipe for.............................................. 89
scab in Australia............................................ 90
vaccine applied to........................................... 188
Slaughter-house examinations........................................ 314

Slee, Henry C.—the physiology of digestion in rumanants............136, 235
some diseases of the foot.................................. 303
Snake bites, a new cure for.................................... 79
Soula, William—reticulated round cell sarcoma of the orbit.............. 60
Splenic fever, meat from cattle suffering from.......................... 75
Stomach, absorption in the dog and cat, rapidity of...................... 81
of the horse, cow, pig and sheep.......................... 81
Strongylus..................................................... 168
Sturgis, A. D.—ulcer of œsophagus............................... 170
Sudor cruentus or blood sweating................................ 57
Swan, asphyxia in a............................................ 264

Teeth of the horse and their diseases.........................143, 288
Temperature of cattle........................................... 84
Tenatomy....................................................... 172
Texas fever.................................................... 190
Trichinæ, do southern hogs have................................. 50
means of detecting.................................... 187
sprialis in the muscles of the hog...................... 83
Trotters for New Zealand....................................... 339
the early development of fast.......................... 278
Turbuculosis, human and bovine.............................252, 266
in cows, the frequency of.....................154, 258, 315
men and animals............................101, 203
Tumors, carcinomatous.......................................... 69

Uterus, inversion of the........................................ 324

Vaccination, protecting animals by.............................. 249
Veterinarians, practical suggestions to young...................... 241
Veterinary anatomist to his dulcinea.............................. 271
bureaus, State.......................................... 49
college, American...................................... 174
Columbia.............................. 173
Edinburgh............................. 268
Montreal.............................. 175
Ontario............................... 175
Congress, the British National........................... 275
degrees, English........................................ 52
department at Harvard................................... 334
honors................................................. 333
Medical Register, supplement to the...................... 99
Society meeting......................... 177
practice, homœopathy in................................. 47
school, new............................................ 194
society, new........................................... 178
surgeons, honors to..................................... 194
royal college of.................... 268

surgical notes........................................ 156
use, syringe for........................................ 265
Virgil as a veterinary poet........................................ 310
Vivisection, practical value of........................................94, 187, 195

Walton, F. V.—caries of a molar tooth........................................ 323
epithelial carcinoma of the left superior maxilla............ 161
reticulated round-cell sarcoma of the mediastinum........ 167
and E. A. MacLellan—a case of colic followed by enteritis.. 259
flatulent colic in a horse............. 260
inspection of the colon in a horse... 260
inversion of the uterus............. 324
lacerated wound in the neck of a horse........................... 261
Wortman, J. L—recent discoveries of fossil horses........................ 281

Zoology, a lesson in comparative........................................ 292
Zoological distribution, a remarkable mode of........................... 78

### BOOKS AND PAMPHLETS RECEIVED.

TWELFTH ANNUAL REPORT OF THE MANHATTAN EYE AND EAR HOSPITAL NEW YORK. 12mo. pp. 48. 1 plate. New York: 1881.

CONTAGIOUS DISEASES OF DOMESTICATED ANIMALS. 8vo. pp. 391. Plates. Washington: 1881.

NINTH ANNUAL MEETING OF THE AMERICAN PUBLIC HEALTH ASSOCIATION. 8vo. pp. 84. Savannah: 1881.

THE CHICAGO MEDICAL JOURNAL AND EXAMINER. Published by the Chicago Medical Press Association. Chicago: 1881.

THE ANNUAL ADDRESS; delivered before the American Academy of Medicine, at its sixth annual meeting in New York, September 20, 1881. By EDWARD T. CASWELL, A.M., M.D., of Providence, R. I. President of the Academy. 8vo. pp. 16. Philadelphia: 1881.

THE GALVANIC ACCUMULATOR; for storing dynamical electricity for cautery and illuminating purposes. By LOUIS ELSBERG, A.M., M.D. 8vo. pp. 13. Cuts. Reprint from the Transactions of the New York Academy of Medicine. New York: 1881.

THE AMERICAN NATURALIST; devoted to the natural sciences in their widest sense. Monthly. Philadelphia: 1881.

NATIONAL LIVE STOCK JOURNAL. Monthly. Chicago: 1881.

THE MEDICAL RECORD: A weekly journal of medicine and surgery. New York: 1881.

THE BLACKSMITH AND WHEELWRIGHT; devoted to the interests of blacksmiths, wheelwrights, carriage and wagon builders, machinists, gunsmiths, and all workers in iron and wood. New York: 1881.

THE BREEDERS' GAZETTE: A weekly journal for the stock-breeder, the turfman, the dairyman, and the general farmer. Chicago: 1881.

THE WESTERN MEDICAL REPORTER: A monthly journal of medicine and surgery. Evansville, Ind.: 1881.

THE COLLEGE AND CLINICAL RECORD: A monthly medical journal. Philadelphia: 1881.

ANNALS AND ANATOMY OF SURGERY: The journal of the Anatomical and Surgical Society of Brooklyn. Brooklyn: 1881.

NASHVILLE JOURNAL OF MEDICINE AND SURGERY. Nashville: 1881.

WALSH'S RETROSPECT; a quarterly compendium of medicine and surgery. Washington: 1881.

VIRGINIA MEDICAL MONTHLY. Richmond: 1881.

THE SOUTHERN CLINIC; a monthly journal of medicine and surgery and new remedies. Richmond: 1881.

NEW ENGLAND MEDICAL MONTHLY. Newtown, Conn.: 1881.

THE CULTIVATOR AND COUNTRY GENTLEMAN. Weekly. Albany: 1881.

INDIANA FARMER; a weekly journal of the farm, home and garden. Indianapolis: 1881.

MIRROR AND FARMER. Weekly. Manchester, N. H.: 1881.

CHICAGO MEDICAL REVIEW. Bi-monthly. Chicago: 1881.

ST. LOUIS CLINICAL RECORD. St. Louis: 1881.

WOCHENSCHRIFT FUR THIERHEILKUNDE UND VIELZUCHT. Augsburg, Germany: 1881.

EL MEDICO Y CIRIYANO CENTRO-AMERICANO. Guatemala, Central America: 1881.

SCHWEIZERITCHES ARCHIV FUR THEIRHEILKUNDE UND THIERZUCHT. Berne Switzerland: 1881.

JOURNAL OF NERVOUS AND MENTAL DISEASES. New York: 1881.

